# 

**Published:** 1846-05

**Authors:** 


### WESTERN JOURNAL

OF

### MEDICINE AND SURGERY:

EDITED BY

DANIEL DRAKE, M. D.

AND

LUNSFORD P. YANDELL, M. D.

PROFESSORS IN THE LOUISVILLE MEDICAL INSTITUTE,

AND

THOMAS W. COLESCOTT, M. D.

---

NO. LXXVII.—MAY, 1846.

---

NEW SERIES.

VOL. V.—NO. V.

---

LOUISVILLE, KY.

PUBLISHED MONTHLY, BY PRENTICE AND WEISSINGER,

PROPRIETORS AND PRINTERS.

1846.

